# Neutrophil-to-lymphocyte ratio and platelet-to-lymphocyte ratio as prognostic markers in patients with extensive-stage small cell lung cancer treated with atezolizumab in combination with chemotherapy

**DOI:** 10.1097/MD.0000000000033432

**Published:** 2023-04-14

**Authors:** Yasin Kutlu, Sabin Goktas Aydin, Ahmet Bilici, Bala Basak Oven, Omer Fatih Olmez, Ozgur Acikgoz, Jamshid Hamdard

**Affiliations:** a Department of Medical Oncology, Medipol University Faculty of Medicine, Istanbul, Turkey; b Department of Medical Oncology, Yeditepe University Faculty of Medicine, Istanbul, Turkey.

**Keywords:** atezolizumab, neutrophil-to-lymphocyte ratio and platelet-to-lymphocyte ratio, small cell lung cancer

## Abstract

Atezolizumab is now the standard treatment for extensive-stage small cell lung cancer (ES-SCLC). Herein, we investigated the prognostic role of inflammatory markers in patients treated with atezolizumab plus chemotherapy and evaluated the efficacy and safety of adding atezolizumab to chemotherapy for patients with ES-SCLC and prognostic and predictive factors as a real-life experience. This retrospective study included 55 patients who received front-line atezolizumab with etoposide plus platin regimen for ES-SCLC. We analyzed the survival outcomes and factors that may predict response and survival. The objective response rate (ORR) was 81.8%. At a median follow-up of 23.5 months, the median progression-free survival (PFS) time was 10.8 months, and the median overall survival (OS) time was 15.2 months. In univariate analysis for PFS, limited-stage disease at the time of diagnosis, the presence of prophylactic cranial irradiation (PCI), the presence of liver metastasis, neutrophil-lymphocyte ratio (NLR), and platelet-lymphocyte ratio (PLR) were found to be prognostic factors (*P* = .041, *P* = .034, *P* = .031, *P* = .004, and *P* = <.001, respectively). In other words, while the median PFS time was 14.1 months in patients with PLR ≤ 135.7, it was 7.5 months in patients with > 135.7. Similarly, median PFS was 14.9 months in patients with NLR ≤ 3.43, while it was 9.6 months in patients with > 3.43. Univariate analysis for OS revealed that limited stage at the time of diagnosis, NLR and PLR were significant prognostic indicators (*P* = .01, *P* = .006, and *P* = .007, respectively). Median OS time for patients with both NLR ≤ 3.43 and PLR ≤ 135.7 was significantly better than that of patients with NLR > 3.43 and PLR > 135.7 (16.9 vs 11.3 and 16.9 vs 11.5 months, respectively). Logistic regression analysis demonstrated that PLR was an independent significant predictive factor for the response to atezolizumab plus chemotherapy (OR: 0.07, *P* = .028). The patients with PLR ≤ 135.7 were significantly good responders to atezolizumab plus chemotherapy treatment. Real-life data demonstrated a significant correlation between survival and NLR and, PLR in ES-SCLC patients treated with atezolizumab. In addition, PLR was a significant predictive indicator of response to atezolizumab plus chemotherapy.

## 1. Introduction

Lung cancer is the most common and deadly cancer worldwide, with approximately 2.1 million new cases of lung cancer and 1.8 million deaths annually. Small cell lung cancer (SCLC) accounts for 15% of all lung cancers and occurs almost exclusively in smokers, and is rare in never smokers.^[1]^ It is a highly aggressive and fatal disease characterized by rapid tumor growth and early distant metastasis, with a 5-year survival rate of <10% at all stages.^[2]^ At the time of diagnosis, approximately 70% of cases present with extensive-stage (ES)-SCLC.^[3]^ However, SCLC is highly sensitive to multiple chemotherapeutic drugs, even in advanced stages.^[4]^

Platinum-based combinations were the standard of care as the initial systemic therapy in patients with extensive-stage small cell lung cancer (ES-SCLC) until the 1990s.^[5]^ Recently, atezolizumab, a humanized monoclonal anti-programmed death-ligand 1 antibody, has been shown to increase survival when combined with carboplatin and etoposide. Therefore, atezolizumab has taken place in front-line treatment.^[6]^ In the IMpower133 phase 3 trial, the addition of atezolizumab or placebo to chemotherapy as first-line treatment for patients with ES-SCLC.^[6]^ The addition of atezolizumab to carboplatin and etoposide resulted in longer overall survival (OS) and progression-free survival (PFS) than chemotherapy alone. The side effects were similar in both groups. Peripheral blood cell analysis is associated with cancer proliferation, invasion, and metastasis. Moreover, the combination of inflammatory indices, such as neutrophil-to-lymphocyte ratio (NLR) and, platelet-to-lymphocyte ratio (PLR), has a significant correlation with many types of cancer survival and prognosis, which has been determined in many trials in the literature.^[7–11]^ High levels of NLR and PLR were significantly associated with shorter OS and PFS and lower response rates in patients with metastatic non-small cell lung cancer treated with nivolumab.^[12]^ However, the association between systemic inflammatory indexes and survival in ES-SCLC treated with atezolizumab plus chemotherapy has not been previously analyzed.

In the present study, we aimed to evaluate the prognostic factors for patients treated with atezolizumab plus chemotherapy, and the safety and efficacy of adding atezolizumab to chemotherapy in patients with ES-SCLC.

## 2. Patients and methods

In this study, a total of 55 patients diagnosed with ES-SCLC between 2016 and 2021, who received front-line atezolizumab with etoposide plus platin regimen, were retrospectively analyzed at the Medipol University Faculty of Medicine. Demographic characteristics such as age, sex, smoking history, ECOG performance score (PS), metastatic sites, response rate, OS, and PFS were evaluated. Inflammatory markers were defined as follows: NLR = (the ratio of neutrophil count to lymphocyte count) and PLR = (the ratio of platelet count to lymphocyte count). The cutoff values for NLR and PLR were determined based on their median index values at the initial diagnosis.^[7,11]^ The association between NLR and PLR and prognosis was also analyzed. The diagnosis of SCLC was confirmed based on histopathology and staged according to the Veterans Affairs Lung Study Group. Limited disease is a tumor confined to the ipsilateral hemithorax, and regional nodes are included. Extensive disease refers to contralateral lung involvement, malignant effusion, and distant metastasis. Patients who could not complete their treatment due to financial problems and non-illness reasons, those who died for reasons other than cancer, and those with ECOG PS 3 and 4 were excluded from the data analysis.

Patients were treated with carboplatin (area under the curve of 5 mg per milliliter per minute, administered intravenously on day 1 of each cycle) and etoposide (100 mg per square meter of body-surface area, administered intravenously on days 1 through 3 of each cycle) with atezolizumab (at a dose of 1200 mg, administered intravenously on day 1 of each cycle).

Patients were evaluated using thoracic CT every 3 cycles. The treatment response, including partial response (PR), complete response (CR), stable disease, and progressive disease, and final objective response rate (ORR: PR and CR) were evaluated according to RECIST 1.1. The study was approved by the local ethics committee of the Istanbul Medipol University.

### 2.1. Statistical analysis

Statistical analyses were performed using SPSS 22.0 (SPSS Inc., Chicago, IL). Descriptive statistics were used to summarize baseline characteristics. Survival analysis was performed using Kaplan–Meier analysis, and comparisons were performed using the log-rank test. PFS was defined as the time from diagnosis until disease progression, date of death, or loss to follow-up. OS described the time from diagnosis to the date of the patient death or loss to follow-up. Univariate analyses were performed to evaluate the significance of NLR, PLR, and other clinicopathological features as prognostic factors. Multivariate analysis with the Cox proportional hazards model was performed to identify independent prognostic factors for both PFS and OS. Predictive factors for response were evaluated using a logistic regression analysis. Hazard ratios were estimated using Cox analysis and reported as relative risks with corresponding 95% confidence intervals (CIs). All *P* values were 2-sided in the tests, and *P* values <.05 were considered statistically significant.

## 3. Results

### 3.1. Clinicopathological characteristics

Forty-two (73%) patients were men and 13 (23.6%) were women with a median age of 63 years (range: 35–83). Three of the patients (5.5%) were nonsmokers, 38 (69.1%) were ex-smokers, and 14 (25.5%) were active smokers. At the initial diagnosis, the majority of patients (89.1%) had extensive-stage disease. Brain metastases were detected in 10 patients (18.2%), and liver metastasis was detected in 19 patients (34.5%) at the initial diagnosis. Thirty-four patients (61.8%) underwent prophylactic cranial irradiation (PCI). While 9.1% of the patients had single-site metastasis, 43.6% had metastasis in 2 sites, 38.2% had metastasis in 3 sites, and 9.1% had metastasis in 4 sites. Patient and tumor characteristics are shown in Table 1.

**Table 1:** Baseline patient characteristics (n = 55).

Characteristics	n (%)
Age, yr (median, range)	63 (35–83)
≤60	23 (41.8)
>60	32 (58.2)
Gender	
Female	13 (23.6)
Male	42 (76.4)
Smoking status	
Never-smoked	3 (5.5)
Ex-smoker	38 (69.1)
Current	14 (25.5)
Stage at diagnosis	
Limited-stage	6 (10.9)
Extensive-stage	49 (89.1)
ECOG PS	
0	29 (52.7)
1	20 (36.4)
2	6 (10.9)
Brain metastasis at diagnosis	
Present	10 (18.2)
Absent	45 (81.8)
No. of metastatic site	
1	5 (9.1)
2	24 (43.6)
3	21 (38.2)
4	5 (9.1)
Liver metastasis	
Present	19 (34.5)
Absent	36 (65.5)
PCI	
Present	34 (61.8)
Absent	21 (38.2)
NLR median, range	3.43 (1.29–21.9)
≤3.43	27 (49.1)
>3.43	28 (50.9)
PLR median, range	135.7 (59.7–827.6)
≤135.7	21 (38.2)
>135.7	34 (61.8)

ECOG PS = Eastern Cooperative Oncology Group performance score, NLR = neutrophil-lymphocyte ratio, PCI = prophylactic cranial irradiation, PLR = platelet-lymphocyte ratio.

### 3.2. Survivals and prognostic factors

The median number of cycles of chemotherapy and atezolizumab was 6 (range: 2–6) and 8 (range: 2–19), respectively. The ORR was 81.8%, while the disease control rate was 90.9%. In 8 patients (14.5%), CR was obtained, while 37 (67.3%) had PR and 5 (9.1%) had stable disease with combination therapy. The ORR to atezolizumab plus combination chemotherapy is shown in Table 2.

**Table 2:** Response rates according to the RECIST 1.1 in ES-SCLC patients treated with atezolizumab in combination with chemotherapy.

Response rate	n (%)
Complete response	8 (14.5)
Partial response	37 (67.3)
Stable disease	5 (9.1)
Progressive disease	5 (9.1)
Objective response rate (CR + PR)	45 (81.8)
Disease-control rate (CR + PR + SD)	50 (90.9)

CR = complete response, ES-SCLC = extensive-stage small cell lung carcinoma, PR = partial response, SD = stable disease.

**Figure 1.**
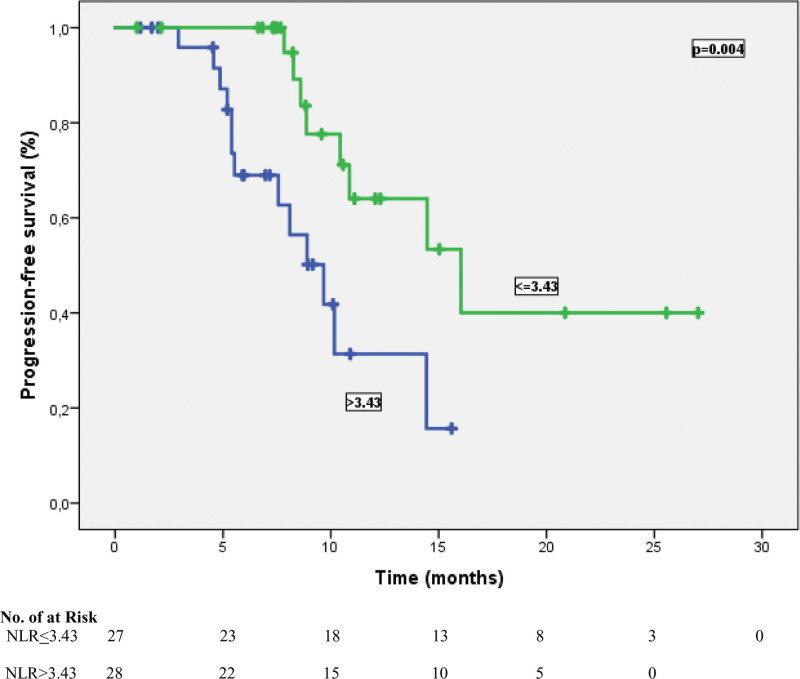
Progression-free survival curve according to NLR status. NLR = neutrophil-lymphocyte ratio.

**Figure 2.**
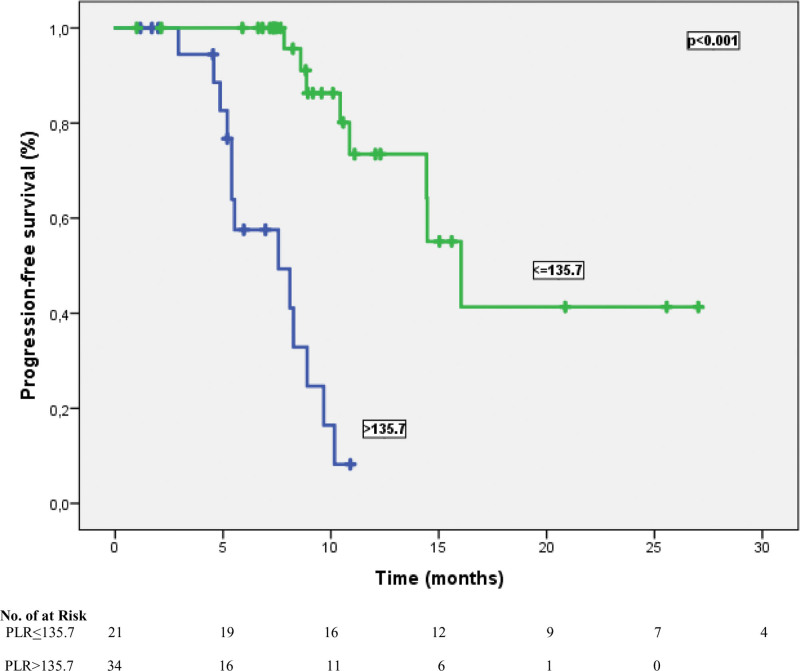
Median progression-free survival for patients with PLR ≤ 135.7 was significantly better than those with ≥135.7. PLR = platelet-lymphocyte ratio.

**Figure 3.**
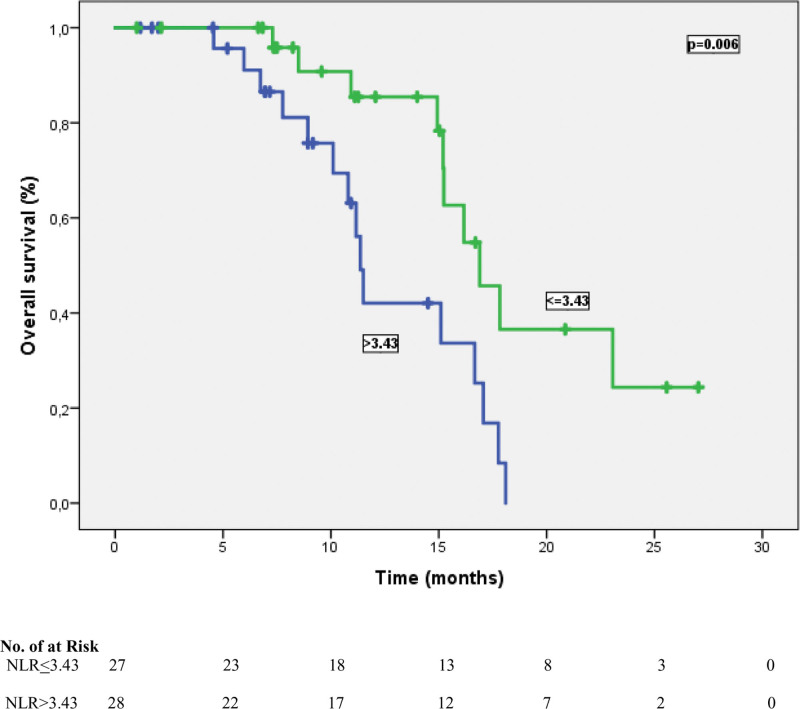
Overall survival outcomes in patients treated with atezolizumab plus chemotherapy according to NLR status (≤3.43 vs ≥3.43). NLR = neutrophil-lymphocyte ratio.

**Figure 4.**
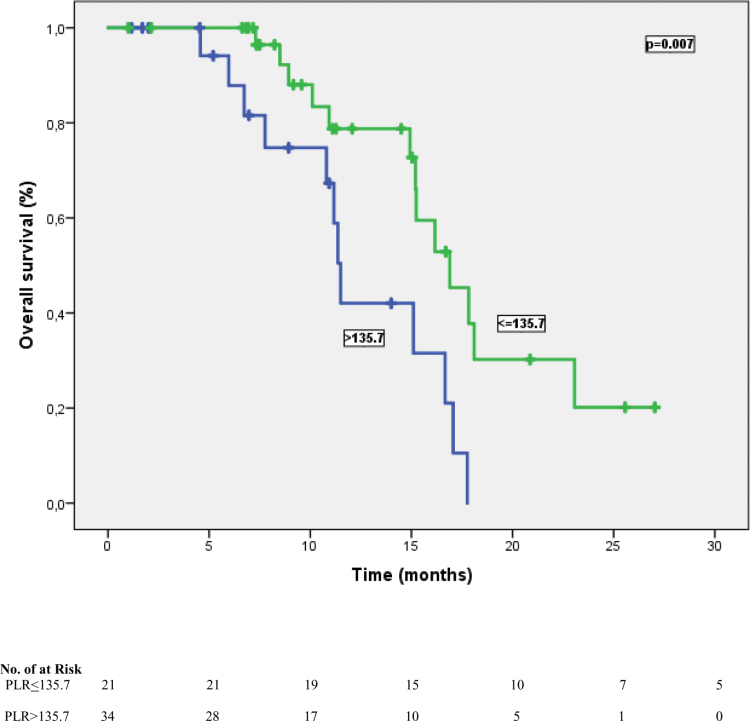
Median overall survival time was worse for patients with PLR ≥135.7 compared to those with PLR ≤135.7. PLR = platelet-lymphocyte ratio.

The cutoff values for NLR and PLR were determined as 3.43 and 135.7, respectively, according to their median. According to this analysis, 27 patients (49.1%) were classified as NLR ≤ 3.43, and 28 patients (50.9%) were classified as NLR > 3.43. In addition, 34 patients (61.8%) were grouped as PLR > 135.7 and 21 patients (38.2%) were categorized as PLR ≤ 135.7. At a median follow-up of 23.5 months, the median PFS time was 10.8 months (95% CI: 5.54–16.1), and the median OS time was 15.2 months (95% CI: 13.5–16.9). In univariate analysis for PFS, limited-stage disease at the time of diagnosis, the presence of PCI, the presence of liver metastasis, NLR, and PLR were found to be prognostic factors (*P* = .041, *P* = .034, *P* = .031, *P* = .004, and P= <.001, respectively). In other words, the median PFS was 14.9 months in patients with NLR ≤ 3.43 and 9.6 months in patients with > 3.43 (*P* = .004) (Fig. 1). Similarly, while the median PFS time was 14.1 months in patients with PLR ≤ 135.7, it was 7.5 months in patients with > 135.7 (*P* = < .001) (Fig. 2). Table 3 shows the results of univariate analysis for PFS. Furthermore, univariate analysis for OS revealed that limited stage at the time of diagnosis, NLR and PLR were significant prognostic indicators (*P* = .01, *P* = .006, and *P* = .007, respectively). The median OS time for patients with NLR ≤ 3.43 was significantly better than that of patients with NLR > 3.43 (16.9 vs 11.3 months, respectively) (Fig. 3). In addition, the median OS time was worse for patients with PLR > 135.7 compared to those with PLR ≤ 135.7 (11.5 vs 16.9 months, respectively) (Fig. 4). The results of univariate analysis for OS are listed in Table 4. In multivariate analysis for PFS, at the time of diagnosis, limited-stage, PCI and PLR were independent prognostic factors, while no significant factors for OS were detected (Tables 3 and 4).

**Table 3:** Univariate and multivariate analysis for progression-free survival (PFS).

Features	Median PFS (mo)	Univariate *P* value	Multivariate *P* value	HR 95% CI
Age, yr		.33		
≤60	10.8			
>60	14.4			
Gender		.73		
Female	10.8			
Male	14.4			
Smoking status		.13		
Never smoked	NA			
Ex-smoker	14.4			
Current smoker	10.8			
Stage at diagnosis		**.041**	**.032**	**3.09 (0.51–9.55**)
Limited-stage	16.0			
Extensive-stage	10.1			
ECOG PS		.68		
0	14.4			
1	8.9			
2	NA			
Brain metastasis at diagnosis		.91		
Present	10.8			
Absent	16.0			
PCI		**.034**	**.034**	**0.34 (0.10–1.08**)
Absent	8.2			
Present	14.4			
No. of metastatic site		.24		
1	14.6			
2	12.9			
3	10.8			
4	NA			
Liver metastasis		**.031**	.65	1.23 (0.48–3.14)
Present	8.1			
Absent	14.4			
NLR		**.004**	.87	1.11 (0.29–4.22)
≤3.43	14.9			
>3.43	9.6			
PLR		**<.001**	**.003**	**0.11 (0.02–0.48**)
≤135.7	14.1			
>135.7	7.5			

Statistically significant values are marked with bold.

CI = confidence interval, ECOG PS **=** Eastern Cooperative Oncology Group performance status, HR = hazard ratio, NA = not applicable, NLR = neutrophil-lymphocyte ratio, NR = not reached, PCI = prophylactic cranial irradiation, PLR = platelet-lymphocyte ratio.

**Table 4:** Univariate and multivariate analysis for overall survival (OS).

Features	Median OS (month)	Univariate *P* value	Multivariate *P* value	HR 95% CI
Age, yr		.80		
≤60	15.1			
>60	16.1			
Gender		.73		
Female	18.1			
Male	15.2			
Smoking status		.27		
Never smoked	NA			
Ex-smoker	16.1			
Current smoker	15.2			
Stage at diagnosis		**.01**	.08	4.18 (0.84–19.7)
Limited-stage	23.0			
Extensive-stage	15.1			
ECOG PS		.34		
0	15.1			
1	17.0			
2	NA			
Brain metastasis at diagnosis		.22		
Present	15.2			
Absent	16.6			
PCI		.06		
Absent	11.3			
Present	16.6			
No. of metastatic site		.83		
1	16.1			
2	16.9			
3	15.1			
4	15.2			
Liver metastasis		.61		
Present	15.2			
Absent	16.6			
NLR		**.006**	.34	0.57 (0.18–1.82)
≤3.43	16.9			
>3.43	11.3			
PLR		**.007**	.58	0.71 (0.21–2.34)
≤135.7	16.9			
>135.7	11.5			

Statistically significant values are marked with bold.

CI = confidence interval, ECOG PS = Eastern Cooperative Oncology Group performance status, HR = hazard ratio, NA = not applicable, NLR = neutrophil-lymphocyte ratio, NR = not reached, PCI = prophylactic cranial irradiation, PLR = platelet-lymphocyte ratio

### 3.3. Predictive factors for response to atezolizumab plus chemotherapy

Logistic regression analysis demonstrated that PLR was an independent significant predictive factor for the response to atezolizumab plus chemotherapy (OR: 0.07, *P* = .028). The patients with PLR ≤ 135.7 were significantly good responders to atezolizumab plus chemotherapy treatment (Table 5).

**Table 5:** Predictive factors for response to atezolizumab in combination with chemotherapy in patients with ES-SCLC.

Factors	Coefficient β	Wald *χ*^2^	*P*	OR	95% CI
NLR	0.66	0.30	.58	1.93	0.18–20.4
PLR	−2.60	4.83	.028	0.07	0.07–0.75
Stage at diagnosis	−1.04	0.88	.34	0.35	0.04–3.09
Liver metastasis	−0.34	0.20	.65	0.70	0.15–3.19
Brain metastasis at diagnosis	−0.77	0.74	.38	0.46	0.07–2.68
No. of metastatic site	0.80	3.02	.08	2.23	0.90–5.54

CI = confidence interval, ES-SCLC = extensive-stage small cell lung cancer, NLR = neutrophil-lymphocyte ratio, OR = odds ratio, PLR = platelet-lymphocyte ratio.

### 3.4. Safety

The most common grade 3/4 adverse events associated with atezolizumab were pneumonitis in 3 patients (8.1%) and, colitis in 1 patient (2.7%). It was not necessary to discontinue treatment because of the side effects. The dose was delayed in 3 of the patients due to side effects. Furthermore, rash (21.6%) and hypothyroidism (18.9%) were common atezolizumab-related grade 1 to 2 adverse events.

## 4. Discussion

Atezolizumab and durvalumab with platinum-based doublet chemotherapy have been shown to improve survival in patients with ES-SCLC and have been approved for the use of front-line treatment. Because of the lack of access to durvalumab in our country, we evaluated the real-life efficacy and safety of atezolizumab as a front-line ES-SCLC treatment.^[13,14]^

The IMpower133 phase 3 trial showed that the addition of atezolizumab to carboplatin and etoposide resulted in significantly longer OS and PFS than chemotherapy alone.^[6]^ The median PFS was 5.2 months in the atezolizumab group and 4.3 months in the placebo group. The median age was 64 years, which is similar to our study. At enrollment, 8.4% of the patients had brain metastasis. However, in our study, 18.2% of the patients had brain metastasis at the time of diagnosis. Despite all these results, we found longer PFS in our study, which may be explained by the small sample size of our study and the median follow-up period of 13.9 months in the IMpower133 study, while our median follow-up period was 23.5 months.

An analysis from China, which evaluated the cost-effectiveness of atezolizumab, included 403 patients.^[15]^ Compared to chemotherapy, atezolizumab significantly improved the PFS (10.3 months vs 12.3 months, respectively). This result is similar to that of our study.^[15]^

When OS was evaluated, the median OS of patients who received atezolizumab in the IMpower133 trial was 13.9 months, while it was 15.2 months in our study. In addition, in the subgroup analysis of the IMpower133 study, the median OS time was also 17.8 months for patients treated with atezolizumab and with high tumor mutational burden (TMB) values.^[6]^ Patients who were given atezolizumab may have had higher TMB scores and, therefore, may have achieved similar survival in this subgroup. We were unable to evaluate the TMB values of the patients in our study.

Qi et al analyzed the systematic inflammatory and nutritional indices in ES-SCLC patients who received atezolizumab plus chemotherapy.^[16]^ They showed a significant correlation between PLR, OS, and PFS. High PLR (>119.23) values were significantly related to poorer prognosis than low PLR values in their study.^[16]^ Similarly, we found that PLR was a significant prognostic factor for both PFS and OS by univariate analysis and a PLR value ≥135.7 was significantly related to worse survival outcomes in our study. However, we could not prove PLR as an independent prognostic indicator in the multivariate analysis. This might be related with the small sample size of our study. Additionally, unlike their study, PLR was also observed as a predictive factor in our study. Thus, patients with PLR ≤ 135.7 were significantly good responders to atezolizumab plus chemotherapy treatment, although our study included a relatively small number of patients. A possible explanation for the predictive significance of PLR and the effectiveness of atezolizumab in addition to chemotherapy may be that carboplatin and etoposide might not deplete the intratumoral T-cell population, and atezolizumab may be able to activate the intratumoral T lymphocytes to exert an antitumor effect. In addition, chemotherapy may increase the efficacy of atezolizumab, possibly by making the tumor microenvironment more immunogenic.

Furthermore, Deng Min et al evaluated the prognostic significance of inflammation markers in SCLC.^[17]^ In this study, similar to our study, the authors demonstrated that NLR was a significant prognostic factor. NLR > 2.65 value was associated with poor prognosis. Similarly, NLR > 3.43 was associated with a poor prognosis in our study. We also investigated NLR as a predictive factor. NLR was not a predictive factor. This might be associated with the small sample size of our study or the addition of atezolizumab to the treatment in our study.

The major limitations of our study were its small sample size and retrospective nature. Moreover, the relatively short follow-up interval was another limitation. These limitations may have affected our findings. We could not analyze predictive molecular markers such as TMB. Although our results should be confirmed by prospective studies, we believe that our study contributes to the literature with respect to real-life analysis of atezolizumab and chemotherapy and shows the potential predictive importance of PLR. Only a few studies have evaluated the efficacy and safety of immunotherapy plus chemotherapy in ES-SCLC. Therefore, our study shows the association of survival with NLR and PLR, as are other clinicopathological factors for patients with ES-SCLC treated with atezolizumab in combination with etoposide and platin in the first-line setting.

In conclusion, NLR and PLR were significant prognostic factors, and atezolizumab plus chemotherapy demonstrated good efficacy and safety profile in ES-SCLC in our real-life analysis. Furthermore, we found that elevated PLR levels were potentially associated with a good response to atezolizumab and chemotherapy, but the importance of NLR as a predictive factor could not be confirmed in predicting response. Future prospective studies with large sample sizes will be needed to address the possible impact of other systemic inflammatory markers on the response to treatment of patients with ES-SCLC. Thus, we might understand who will benefit from treatment.

## Author contributions

**Conceptualization:** Bala Basak Oven.

**Data curation:** Sabin Goktas Aydin, Omer Fatih Olmez, Ozgur Acikgoz, Jamshid Hamdard.

**Formal analysis:** Ozgur Acikgoz.

**Investigation:** Omer Fatih Olmez, Jamshid Hamdard.

**Methodology:** Ahmet Bilici, Bala Basak Oven, Omer Fatih Olmez.

**Project administration:** Ahmet Bilici, Omer Fatih Olmez.

**Resources:** Ahmet Bilici, Bala Basak Oven, Ozgur Acikgoz.

**Validation:** Bala Basak Oven.

**Visualization:** Ozgur Acikgoz.

**Writing – original draft:** Yasin Kutlu.

**Writing – review & editing:** Sabin Goktas Aydin, Ahmet Bilici, Omer Fatih Olmez.
